# Utility of oxygen insufflation through working channel during fiberoptic intubation in apneic patients: a prospective randomized controlled study

**DOI:** 10.1186/s12871-020-01201-9

**Published:** 2020-11-10

**Authors:** Go Un Roh, Joon Gwon Kang, Jung Youn Han, Chul Ho Chang

**Affiliations:** 1 Department of Anesthesiology and Pain Medicine, CHA Bundang Medical Center, CHA University School of Medicine, 59 Yatap-ro, Bundang-gu, Seongnami-si, Gyeonggi-do 13496 Korea; 2 Department of Anesthesiology and Pain Medicine, and Anesthesia and Pain Research Institute, Yonsei University College of Medicine, Gangnam Severance Hospital, 211 Eonju-ro, Gangnam-gu, Seoul, 06273 Korea

**Keywords:** Fiberoptic intubation, Apneic oxygenation, Oxygenation during intubation

## Abstract

**Background:**

Airway management is a part of routine anesthetic procedures; however, serious complications, including hypoxia and death, are known to occur in cases of difficult airways. Therefore, alternative techniques such as fiberoptic bronchoscope-assisted intubation (FOB intubation) should be considered, although this method requires more time and offers a limited visual field than does intubation with a direct laryngoscope. Oxygen insufflation through the working channel during FOB intubation could minimize the risk of desaturation and improve the visual field. Therefore, the aim of this prospective randomized controlled study was to evaluate the utility and safety of oxygen insufflation through the working channel during FOB intubation in apneic patients.

**Methods:**

Thirty-six patients were randomly allocated to an N group (no oxygen insufflation) or an O group (oxygen insufflation). After preoxygenation, FOB intubation was performed with (O group) or without (N group) oxygen insufflation in apneic patients. The primary outcome was the velocity of decrease in the partial pressure of oxygen (PaO_2_) during FOB intubation (V_PaO2_, mmHg/sec) defined as the difference of PaO_2_ before and after intubation divided by the time to intubation. The secondary outcomes included the success rate for FOB intubation, time to intubation, visual field during FOB intubation, findings of arterial blood gas analysis, and occurrence of FOB intubation-related complications.

**Results:**

We found that V_PaO2_ was significantly greater in the N group than in the O group (1.0 ± 0.4 vs. 0.4 ± 0.4; *p* < 0.001), while the visual field was similar between groups. There were no significant intergroup differences in the secondary outcomes.

**Conclusions:**

These findings suggest that oxygen insufflation through the working channel during FOB intubation aids in extending the apneic window during the procedure.

**Trial registration:**

ClinicalTrials.gov, NCT02625194, registered at December 9, 2015.

## Background

Airway management is a part of essential anesthetic procedures. However, it becomes challenging in cases of difficult airways and may cause several complications, including airway trauma, hypoxia, and even death [1, 2]. Therefore, intubation techniques involving the use of fiberoptic bronchoscopes (FOBs) and videolaryngoscopes should be considered as alternative options [3, 4]. During FOB-assisted intubation (FOB intubation), superior visualization of anatomical structures is the key for successful intubation, and suction through the working channel of FOB is commonly performed for the clearance of secretions and blood and improvement of the visual field. Oxygen insufflation through this working channel during the procedure is also possible and recommended by some physicians [5–7]. Oxygen insufflation through the working channel during FOB intubation, which requires a longer time than does direct laryngoscopic intubation, can minimize the risk of desaturation in apneic patients [8]. In addition, oxygen flow at the end of FOB could help in the clearance of secretions and small structural barriers, resulting in improved visualization during the procedure [5, 9]. However, a few reports have documented rare but critical complications such as gastric rupture and pneumothorax [10–12]. Moreover, this technique is not recommended for pediatric patients or patients with significant airway edema [11]. Thus, oxygen insufflation through the working channel during FOB intubation has certain advantages and limitations. However, few studies have evaluated its utility and safety in clinical practice. Therefore, the aim of the present study was to evaluate the utility and safety of oxygen insufflation through the working channel during FOB intubation via measurement of the velocity of decrease in the partial pressure of oxygen (PaO_2_) during the procedure (V_PaO2_)_._ In addition, the time to intubation, visual field, and occurrence of FOB intubation-related complications were investigated.

## Methods

### Patients

This prospective randomized controlled study was approved by the Institutional Review Board of Yonsei University Gangnam Severance Hospital (IRB No 3–2015-0218, NCT02625194, https://clinicaltrials.gov/ct2/show/NCT02625194?term=NCT02625194&rank=1). After the acquisition of written informed consent, 36 patients aged 20–60 years (American Society of Anesthesiolgists class I to III) who were scheduled for elective surgery under general endotracheal anesthesia with arterial cannulation at Yonsei University Gangnam Severance Hospital were included. The exclusion criteria included lung disease, anticipated difficult airway, inability to read or write, and pregnancy. The randomization table at www.random.org was used to allocate patients to an N group (no oxygen insufflation) or an O group (oxygen insufflation) at ratio of 1:1.

### Study design and procedure

After premedication with 0.02 mg/kg of midazolam and 0.004 mg/kg of glycopyrrolate in the preanesthesia care unit, patients were transferred to the operating room, where standard monitoring procedures, including electrocardiography, pulse oximetry, and noninvasive blood pressure measurement, were initiated. Vital signs, including the heart rate, systolic blood pressure, diastolic blood pressure, and peripheral oxygen saturation, were recorded before anesthesia induction. Preoxygenation with 100% oxygen using a facemask was performed for 5 min, following which anesthesia was induced with propofol 1.5 mg/kg, remifentanil 0.1 mcg/kg/min, and rocuronium 0.8 mg/kg. The radial artery was cannulated during manual ventilation. After 2 min of manual ventilation, arterial blood was collected and the facemask was removed for intubation (T1). The airway was opened by the jaw thrust maneuver, and endotracheal intubation was performed using a flexible FOB (Intubation fiberscope, 5.2 × 65 mm, Karl Stortz GmbH & CO, Germany) with a preloaded endotracheal tube. In the O group, 100% oxygen was administered at 5 L/min through the working channel. The N group did not receive oxygen insufflation. Intubation success was confirmed by bronchoscopy, following which arterial blood was collected before the initiation of ventilation (T2). The first end-tidal carbon dioxide value after the resumption of ventilation following intubation was recorded.

### Study endpoints

The primary endpoint was V_PaO2,_ defined as the difference of PaO_2_ between T1 and T2 divided by the time to intubation (mmHg/sec). Secondary endpoints included the FOB intubation success rate, time to intubation (from facemask removal until resumption of ventilation), visual field during FOB intubation (excellent, clear view of anatomical structures and no limitation; good, < 50% limitation in the view but no difficulty in intubation; poor, > 50% limitation in the view, resulting in significant difficulty in intubation; and impossible, inability to identify the anatomical structures and intubate), and occurrence of FOB intubation-related complications, including mucosal injury, abdominal distension, postoperative nausea and/or vomiting, and desaturation [peripheral capillary oxygen saturation (SpO_2_) < 90%]. In addition, vital signs and pH, partial pressure of carbon dioxide (PaCO_2_), and PaO_2_ values were recorded for all patients.

### Statistical analysis

Considering the findings of Rosenstock, who reported a mean V_PaO2_ of 0.9 mmHg/s with a standard deviation of 0.17, we found that 16 patients per group were required for detection of a 0.25 mmHg/s decrease in V_PaO2_ with a type 1 error and power of 0.05 and 80%, respectively [13]. Accordingly, a total of 18 patients per group were included after accounting for the withdrawal rate during study. All statistical analyses were performed using SPSS, version 18.0 (SPSS Inc., Chicago, IL, USA). For V_PaO2_, Mann-Whitney U test was performed. Secondary endpoints including time to intubation, vital signs and arterial blood gas analysis data were also assessed with Mann-Whitney U test. Visual field and FOB intubation-related complications were analysed by Fisher’s exact test. In comparing the patients’ characteristics, continuous variables were assessed using Mann–Whitney U-test, while categorical variables were assessed using the chi-square test or Fisher’s exact test. The results from Mann-Whitney U test were expressed as mean with standard deviation. The categorical data was presented as number. The Bonferroni method was used to compensate the error of multiple comparisons. A *P*-value of < 0.05 was considered statistically significant.

## Results

**Fig. 1:**
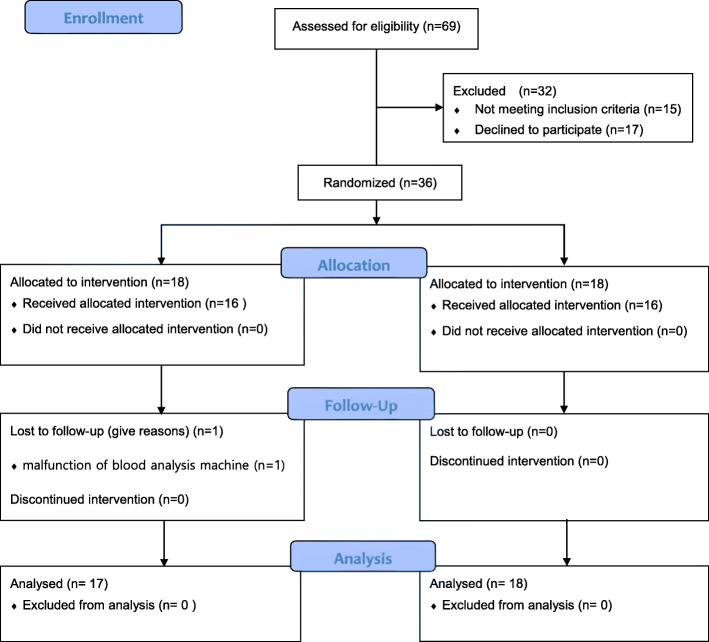
CONSORT diagram showing the flow of participants

In total, 36 patients were enrolled, 35 of whom completed the study. One patient in the N group was excluded because of malfunction of the arterial blood gas analysis machine (Fig. 1). The patients’ baseline characteristics were not different between the two groups (Table 1). All patients were successfully intubated. Vital signs and pH, PaCO_2_, PaO_2_ and SaO_2_ values also showed no significant intergroup differences throughout the procedure. The time to intubation was approximately 150 s in both groups. Although PaO_2_ showed no significant intergroup differences at T1 and T2, the decrease in PaO_2_ during FOB intubation was significantly greater in the N group than in the O group (139.4 ± 74.3 vs. 72.0 ± 67.7, *p* = 0.012). In addition, V_PaO2_ was significantly greater in the N group than in the O group (1.0 ± 0.4 vs. 0.4 ± 0.4, *p* < 0.001). An excellent visual field was obtained for 12 and 7 patients in the O and N groups, respectively, with no significant intergroup difference. None of the patients presented mucosal injury, abdominal distension, and desaturation. One patient in the O group exhibited postoperative nausea (Table 2).

**Table 1:** Patients’ Characteristics

	No Oxygen (*n* = 17)	Oxygen (*n* = 18)	*P*-value	Standardized differences
Age (year)	53.1 ± 15.7	59.1 ± 9.6	0.330	0.622
Sex (male, number)	5 (29.4%)	6 (33.3%)	0.803	0.083
Height (cm)	161.6 ± 10.7	162.4 ± 9.1	0.655	0.094
Weight (kg)	63.9 ± 12.5	66.1 ± 14.0	0.741	0.159
ASA Class			0.391	0.581
I	8 (47.1)	4 (22.2)		
II	8 (47.1)	12 (66.7)		
III	1 (5.9)	2 (11.1)		

Values are expressed as mean ± standard deviation or number

*ASA* American Society of Anesthesiologist

**Table 2:** Primary and secondary endpoints

	No Oxygen (*n* = 17)	Oxygen (*n* = 18)	*P*-value
Heart rate (bpm)			
T1	75.1 ± 15.5	67.9 ± 10.4	0.291
T2	93.4 ± 15.9	87.2 ± 14.9	0.261
SBP (mmHg)			
T1	105.2 ± 21.7	106.1 ± 17.4	0.428
T2	142.4 ± 33.1	132.1 ± 27.5	0.391
DBP (mmHg)			
T1	57.8 ± 12.0	61.9 ± 14.5	0.437
T2	79.9 ± 19.6	77.8 ± 18.1	0.656
SpO_2_ (%)			
T1	99.4 ± 0.7	99.4 ± 0.9	0.809
T2	99.0 ± 1.3	98.7 ± 1.2	0.376
pH			
T1	7.4 ± 0.1	7.4 ± 0.0	0.106
T2	7.3 ± 0.0	7.3 ± 0.0	0.373
SaO_2_			
T1	99.9 ± 0.2	99.9 ± 0.1	0.929
T2	99.4 ± 1.2	99.6 ± 1.4	0.335
PaCO_2_			
T1	37.6 ± 6.4	38.3 ± 6.1	0.552
T2	46.7 ± 6.6	47.5 ± 6.1	0.632
PaO_2_ (mmHg)			
T1	415.0 ± 90.4	379.7 ± 72.3	0.137
T2	275.6 ± 94.9	307.8 ± 97.2	0.322
PaO_2_ difference (mmHg)	139.4 ± 74.3	72.0 ± 67.7	0.012
Intubation time (sec)	145.5 ± 74.8	155.9 ± 70.1	0.632
VPaO_2_ (mmHg/sec)	1.0 ± 0.4	0.4 ± 0.4	0.000
Visual field			0.183
Excellent	7	12	
Good	8	3	
Poor	2	3	
Complication			
PONV (N)	0	1	1.000

Values are expressed as mean ± standard deviation or number

*T1*, 2 min after manual ventilation with 100% oxygen, *T2* after intubation confirmed with bronchoscopy, before ventilation resumed, *SBP* systolic blood pressure, *DBP* diastolic blood pressure, *SpO*_*2*_ peripheral oxygen saturation, *SaO*_*2*_ arterial oxygen saturation, *PaCO*_*2*_ arterial partial pressure of carbon dioxide, *PaO*_*2*_ arterial partial pressure of oxygen, *PaO*_*2*_ *difference* difference of arterial partial pressure of oxygen between T1 and T2, *VPaO*_*2*_ velocity of decrease in arterial partial pressure of oxygen, *PONV* postoperative nausea and vomiting

## Discussion

In the present study assessing the utility of oxygen insufflation during FOB intubation, V_PaO2_ was significantly reduced when oxygen was insufflated through the working channel. However, patients with and without oxygen insufflation showed no significant differences in the intubation success rate, time to intubation, visual field, and occurrence of complications.

Despite recent advances in airway management devices such as videolaryngoscopes and supraglottic airways, FOB intubation is still considered the gold standard for difficult airway management [14, 15]. However, it requires a longer time than does intubation with a direct laryngoscope. In addition, secretions and bleeding in the airway obscure the airway anatomy and complicate intubation [3]. Therefore, methods to improve patient oxygenation after the induction of anesthesia can facilitate faster and safer FOB intubation.

Several studies have attempted to overcome the limitations of FOB intubation. A direct laryngoscope or GlideScope can assist the placement of FOB near the glottis and facilitate easy intubation [16–18]. In addition, supraglottic airways such as I-gel or LMA can be used as conduits for FOB intubation, providing a better route to the glottic inlet and, at the same time, ventilating the lungs during the procedure [19, 20]. However, these techniques require the use of additional devices and result in an increased time to intubation. Especially, they are inappropriate in patients with limited mouth opening [21]. Oxygen insufflation through the working channel during FOB intubation does not require extra time and devices. Oxygen insufflation through the working channel of FOB could alleviate both hypoxia and visual field impairment in children [5]. In our study, V_PaO2_ was 1.01 ± 0.39 mmHg/s in the N group, which was more than twice the value for the O group (0.42 ± 0.42 mmHg/s). If the hypoxia-free apnea time during FOB intubation is defined as the time from the discontinuation of mask ventilation at a PaO_2_ of 500 mmHg to the achievement of 90% SpO_2_ at a PaO_2_ of 60 mmHg, under the assumption that PaO_2_ exhibits a linear decrease during apnea, the hypoxia-free apnea duration could be approximately 10 min longer in the O group than in the N group (1047.62 s vs. 435.64 s, respectively).

This calculation is based on a ventilatory mass flow, also known as apneic oxygenation. During regular breathing in an adult, oxygen and carbon dioxide are exchanged between the lungs and blood at a flow rate of 250 ml/min. During apnea, the carbon dioxide flow returning to the lungs is significantly reduced to 8–20 ml/min while the oxygen flow to the blood is maintained. Therefore, negative pressure is generated in the lungs according to the volume difference during oxygen and carbon dioxide exchange; this facilitates the movement of oxygen from the pharynx to the lungs [8, 22].

Apneic oxygenation could be applied in various clinical situations involving different types of devices, including nasal prongs, nasopharyngeal catheters, and tracheal or bronchial catheters. The first two are commonly used because they are practical [8]. Oxygenation with nasal prongs at 5 l/min during FOB intubation could lower the decrease in PaO_2_ at 3 min during apnea [23]. In Teller’s crossover study evaluating the influence of oxygen delivery via a nasopharyngeal catheter at 3 l/min, apnea was continued for 10 min or until SpO_2_ decreased to 92%. None of the patients in the apneic oxygenation group showed an SpO2 of < 97% until 10 min. On the other hand, the mean apnea time in the control group was 6.8 min and the lowest SpO_2_ value was 91% [24]. In present study, we used the working channel of FOB to continuously deliver oxygen as FOB moved toward the trachea. This increases the efficiency of oxygen delivery to the lungs and could be particularly useful for patients prone to desaturation during apnea, such as obese patients [25].

One limitation of apneic oxygenation is that it cannot efficiently remove carbon dioxide from the blood, resulting in an increase in the carbon dioxide level at a rate of 1.1–3.4 mmHg/min and, eventually, hypercarbia and acidosis [8]. Therefore, it should not be used in patients with a risk of hypercarbia-related complications. Of late, the use of a high flow nasal cannula has garnered attention in various clinical situations, and it can be effectively used for apneic oxygenation during intubation as well. Studies found that it could deliver a high concentration of oxygen, generate a positive airway pressure of approximately 7 cmH_2_O, and slow down the increase in the carbon dioxide level during apneic oxygenation [22, 26]. In the present study, the increase in PaCO_2_ during the apneic period was similar in the O and N groups.

With regard to the visual field, the number of patients with an excellent visual field was not significantly different between groups, although the number was higher in the O group. This result was inconsistent with those of previous reports, and there are a few possible explanations for the discrepancy. Unlike Rosen’s study, which included pediatric patients with difficult airways and involved the use of smaller FOBs [5], the present study included adult patients without anticipated difficult airways and involved the use of FOBs with a larger diameter. Moreover, all patients were premedicated with glycopyrrolate in order to minimize secretions [3]. The visual field can be easily disrupted during awake FOB intubation because of patient movement and lens fogginess caused by spontaneous breathing [7]. However, we performed the study under anesthesia with complete muscle relaxation, so there were no disturbances during FOB intubation. Therefore, the intubation conditions were quite good, and the actual effect of oxygen insufflation may not have been as significant as expected.

The two groups in our study showed a comparable intubation success rate and time to intubation; this could be attributed to the lack of differences in the visual field quality. The optimal intubation conditions may be another factor that repressed the influence of oxygen on the intubation-related parameters. We believe that different results may be derived if the measurements were recorded in emergency situations involving patients with unanticipated difficult airways. Further studies should take this aspect into consideration and assess the usefulness of oxygen insufflation during FOB intubation in different clinical scenarios.

From our results, it is evident that oxygen insufflation through the working channel of FOB can reduce VPaO_2_ during FOB intubation. Oxygen insufflation through the working channel of FOB can cause rare but serious complications such as gastric rupture and pneumothorax [10, 11, 27]. Oxygen can enter the trachea or esophagus during the procedure. If oxygen is delivered to the esophagus and stomach, it could cause nausea, vomiting [10, 12]. If FOB with oxygen insufflation enters the stomach, it could cause significant distension of the stomach and even rupture, because the maximal capacity of the stomach is approximately 1 l, and FOB could deliver approximately 2.5 l of oxygen in just 30 s, theoretically [10, 28]. However, according to Wong’s review of several studies on nasal or nasopharyngeal apneic oxygenation, no complications related to pressure effects have been reported till date [8]. The esophagus is closed by a sphincter, and approximately 20 cmH_2_O of pressure is required to open it [29]. According to a previous report using nasal high flow oxygen insufflation, the mean airway pressure was approximately 7 cmH_2_O [26], which is considerably lower than the pressure required to open the esophageal sphincter. In the present study, none of the patients complained of vomiting or abdominal distension, and only one patient in group O developed postoperative nausea. Furthermore, there was no case of mucosal injury or desaturation during the procedure. This was probably due to the optimal intubation conditions and the completion of intubation within 5 min, which is considered safe if the patient is preoxygenated [26].

This study has some limitations. First, FOB intubation was conducted under optimal conditions as described earlier. Therefore, the results cannot be applied to other clinical situations such as emergency difficult airway management. Second, our sample size was enough for VPaO_2_ measurement but not adequate to assess the occurrence of complications, which are anyways rare.

## Conclusions

In conclusion, our findings suggest that oxygen insufflation through the working channel of FOB can help in extending the apneic window after the induction of general anesthesia during FOB intubation, with minimal complications.

## Data Availability

The datasets used or analysed during the current study are available from the corresponding author on reasonable request.

## Abbreviations

FOB intubation: Fiberoptic bronchoscope-assisted intubation
FOB: Fiberoptic bronchoscope
PaO_2_: Partial pressure of oxygen
V_PaO2_: Velocity of decrease in the partial pressure of oxygen
PaCO_2_: Partial pressure of carbon dioxide
SpO_2_: Peripheral capillary oxygen saturation
